# Fingerprinting of Doppler audio signals from the common carotid artery

**DOI:** 10.1038/s41598-020-59274-y

**Published:** 2020-02-12

**Authors:** Anna V. Müller, José M. Amigo, Nicoline R. Wichmann, Frederik B. Witschas, Fintan J. McEvoy

**Affiliations:** 1 Department of Veterinary Clinical Sciences, University of Copenhagen, Dyrlægevej 16, DK-1870 Frederiksberg C, Denmark; 2 Department of Science, Spectroscopy and Chemometrics, University of Copenhagen, Rolighedsvej 26, DK-1958 Frederiksberg, Denmark; 3 Present Address: Ikerbasque, Basque Foundation for Sciences, Maria Díaz de Haro 3, Bilbao, 48013 Spain; 4 Present Address: Department of Analytical Chemistry, University of the Basque Country, Barrio Sarriena S/N, Leioa, 48940 Spain

**Keywords:** Ultrasonography, Translational research

## Abstract

Audio fingerprinting involves extraction of quantitative frequency descriptors that can be used for indexing, search and retrieval of audio signals in sound recognition software. We propose a similar approach with medical ultrasonographic Doppler audio signals. Power Doppler periodograms were generated from 84 ultrasonographic Doppler signals from the common carotid arteries in 22 dogs. Frequency features were extracted from each periodogram and included in a principal component analysis (PCA). From this 10 audio samples were pairwise classified as being either similar or dissimilar. These pairings were compared to a similar classification based on standard quantitative parameters used in medical ultrasound and to classification performed by a panel of listeners. The ranking of sound files according to degree of similarity differed between the frequency and conventional classification methods. The panel of listeners had an 88% agreement with the classification based on quantitative frequency features. These findings were significantly different from the score expected by chance (p < 0.001). The results indicate that the proposed frequency based classification has a perceptual relevance for human listeners and that the method is feasible. Audio fingerprinting of medical Doppler signals is potentially useful for indexing and search for similar and dissimilar audio samples in a dataset.

## Introduction

Audio fingerprinting is inspired by the human fingerprint, which functions as a small but unique representation of a person. The purpose of audio fingerprinting is to construct a quantitative, digital summary of an audio signal, which can then be used for matching and searching among large numbers of signals^1^. Search in this context refers to either finding an exact match to a given sound, or finding sounds that have similar features. Thus the presence of an exact match or the degree of similarity can be determined by a search. Fingerprinting has been applied to audio signals in speech recognition software, in music and environmental sound identification, allowing search and retrieval of identical or similar audio signals from databases^1–3^. In medicine, similar applications are far less plentiful, although automatic analysis of respiratory, heart and bowel sounds have been explored^4–6^. In medical imaging, Doppler ultrasound is used to quantify blood flow. The technique utilizes the Doppler effect of frequency shift as the sound is reflected from moving objects. Flow velocity and acceleration is processed and displayed by the ultrasound machine^7^. These blood flow data are presented in two ways simultaneously; a graphical spectral display of the Doppler shift (velocity) against time, and a real time continuous audio signal that will change in rhythm, frequency and amplitude according to normal cyclical alterations in blood flow. A number of widely used parameters are typically extracted from the graphical display^8^. This is done automatically by the ultrasound equipment. They are all time based parameters or ratios of such parameters and are described in detail in the methods. Objective analysis of the actual Doppler sounds in the frequency domain has been reported, but is not common practice in medical imaging^9–11^. In fact, the Doppler audio signal is rarely recorded. This is unfortunate as a systematic search of an indexed database of audio files and their associated patient diagnosis and outcome would be a useful resource to clinicians in their decision making process.

The aim of this study is to create a dataset of arterial Doppler sounds and then use an audio fingerprinting method to index the samples and identify similar and dissimilar samples within the dataset. We describe the extraction of frequency features from the signal using common spectral analysis techniques. Similarities and dissimilarities among the extracted features are identified using principal component analysis (PCA). The validity of the results is tested against similar classifications made by subjective human evaluation. The novelty of the analysis is tested by a comparison with a classification based on conventional medical Doppler parameters. We propose that audio signal fingerprinting has a role in medical ultrasound, as it provides the potential to allow efficient indexing and search, and can contribute to patient care.

## Material and Methods

### Study population

The study was carried out in a veterinary teaching hospital setting. Healthy dogs were recruited within personal networks of staff and students, at the University Hospital for Companion Animals at the University of Copenhagen. No selection was made for breed, age, sex or size. All dogs underwent a clinical examination, including auscultation of heart and lungs and were found to be normal.

### Doppler ultrasonography

**Figure 1:**
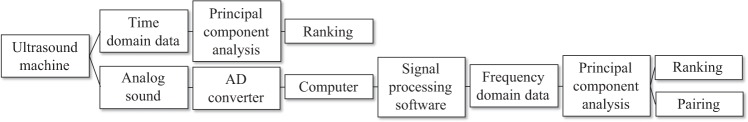
Flowchart showing two data-streams taken from the medical ultrasound machine. The ultrasound machine has two outputs, time domain data parameters and an analog signal which is recorded. The time domain data was available from the machine and was included directly in a principal component analysis that allowed ranking of the time domain data according to the degree of similarity. The analog signal is first digitized in an analog digital converter (AD converter). The resulting digital data is exported to a computer where the signal processing software performs a Fourier transform to extract the frequency domain data. These data are included in another principal component analysis that allowed the identification of similar and dissimilar pairs and also a ranking of the sound files according to the degree of similarity.

Doppler ultrasound data, in the form of color flow mapping images, spectral displays and audio recordings were collected from the right and left common carotid arteries in each dog. The scanning technique required that a region of the coat was clipped on each side of the mid-third of the ventral neck. Alcohol (70%) and coupling gel was applied to the skin. The ultrasound examinations were performed using a GE LOGIQ E9 scanner with a 5–9 MHz transducer (GE Volusion RNA). Duplex pulsed wave Doppler was used with a gate size of 1 mm, which corresponded to 1/3–1/2 of the common carotid artery lumen diameter in most dogs. The insonation angle was maintained at or below 60°. A flowchart of the data acquisition, processing and analysis is given in Fig. 1. These steps are described in detail below.

### Color flow maps and spectral display acquisition

When the pulsed waveforms from at least 5 cardiac cycles were distinctly identified on the screen, the image was frozen and the ultrasound software automatically calculated and displayed peak-systolic velocity (PSV), end-diastolic velocity (EDV), resistive index (RI) and pulsatility index (PI). These data and the image of the spectral display were saved to the ultrasound machine’s disk drive. This process was repeated so that 6 samples were obtained from each vessel. The entire process resulted in multiple sets of conventional Doppler parameters for each vessel. These parameters are described in the signal processing section below.

### Audio acquisition

The ultrasound machine was coupled via two audio line out RCA connectors to a combined pre-amplifier and analog to digital converter (Focusrite Scarlett 2i2, Focusrite Audio Engineering Ltd., High Wycombe, Bucks, UK). The converter in turn was connected via a USB connection to a computer (MacBook Pro, OS X Yosemite 10.10.5, Apple Inc., Cupertino, CA, USA) running a commercial digital audio workstation (GarageBand 10.1.4, Apple Inc., Cupertino, CA, USA). Four arterial Doppler audio signals from the carotid artery were recorded, two from the right and two from the left common carotid arteries. In two dogs, two recordings only were acquired. The audio recording was optimized by adjusting the gain of the digital audio workstation manually during each recording. Each audio signal contained a minimum of 8 cardiac cycles and was saved in Audio Interchange File (.aif) format to the laptop computer.

### Signal processing

Analysis was performed using commercial software (MATLAB version 2014a; The Mathworks Inc., Natick, MA) and the Signal Processing toolbox for Matlab.

### Spectral analysis in frequency domain

Each audio file was opened in MATLAB. These files contain a large vector of amplitude values and a single scalar to indicate the sampling frequency used to create the file. This latter value was 44100 Hz for all recordings used in this study. These file parameters, amplitude and sampling frequency, were used to obtain a periodogram, which is a power spectral density estimate. This periodogram gives the power (dB) of the sound for each frequency in the signal. It was achieved using the “periodogram” function in Matlab, which applies the discrete Fourier Transform to the data and outputs the amplitude of the frequencies in the signal. Parameters were set so that the frequency vector returned contained 100,000 frequency values. These were further binned, each bin containing amplitude data for 80 frequencies (1250 bins) presented as the mean of the data in each bin. Doppler power (amplitude vs. frequency) spectra usually also include low-frequency signals from the vessel wall, and high-frequency noise caused by the instrumentation^12^. Therefore, the lowest and highest frequencies were excluded by selecting a frequency range from the 10^th^ to the 200^th^ bin. These data when plotted (power against frequency) produced a spectrum with well-defined peaks and troughs. This plot displays the frequencies encountered and their amplitude during the evaluation period, which was a minimum of 8 spectral waveforms. The 10 most prominent peaks in the spectrum were automatically detected, using the “findpeaks” function in MATLAB. For each of these selected peaks 4 standard signal processing parameters were extracted. These were

(1) the peak prominence

(2) the height of the peak

(3) the width of the peak at half prominence

(4) the location (mean frequency for the bin in which the particular peak occurred).

This resulted in a total of 4 parameters for each of the 10 peaks from each sound file (i.e. 40 values per sound file).

The set of values for each of these parameters was then normalized so that the maximum value for each parameter was one. These 40 values for each audio sample in the normalized data set were then reduced to the following 5 parameters:

(1) the sum of the peak prominences

(2) the sum of the peak heights

(3) the sum of the peak widths at half prominence

(4) the mean of the peak locations

(5) the variance of the peak locations.

Each of these 5 parameters is represented as a single value that was again normalized across all audio files, to a maximum of one. Each of the five parameters was used in the PCA. In this way each sound file is represented by a vector of length 5, whose values are derived from frequency analysis. These 5 features are referred to as frequency domain features in the following text.

### Spectral analysis in time domain

Four of the features that were automatically extracted by the default machine software were recorded for each vessel. These were: peak systolic velocity (PSV), end diastolic velocity (EDV), pulsatility index (PI) and resistive index (RI). PI is the difference between PSV and the minimum velocity divided by the mean velocity and RI is the difference between PSV and EDV divided by PSV^13^. These four features will be referred to as time domain features in the following text. The values reported here for these features are means for each vessel from six selected samples. Thus two means of each conventional flow feature (one from the left and one from the right carotid artery) were calculated for each dog, and used in further statistical analysis.

## Statistical Tests

### Descriptive statistics

Descriptive statistics were generated for the conventional Doppler data. The standard deviation was used as an indicator of variation.

### Multivariate analysis

PCA is a non-supervised machine learning method based on co-variance. PCA identifies axes in the data, where the variance among samples is maximized. Those axes are the principal components (PCs). The first PC (PC1) explains the most variance, the second PC (PC2) explains the second most variance etc., with the constraint that each PC is perpendicular to the previous one^14^. The analysis results in scores representing the samples, and loadings representing the variables, which can be explored visually in a scatter plot. In a plot of the scores, proximity or clustering of the scores indicates similarity between samples. Such similarity is with regard to those features, which have similar influence (loadings) on the variance for the given samples. Scores that are relatively remote from each other indicate dissimilarity between samples, with regard to those features which have widely different influence (loadings) on the variance of the given samples. On the loadings plot, features with loading values far from zero have a greater influence than loadings in the center of the plot^15^. It is emphasized that the PCA model does not identify why scores are similar or dissimilar, that is for the operator to explore. The five frequency domain features extracted from each audio signal were included in a PCA. The four time domain spectral features in time domain (PSV, EDV, PI, RI) were included in a separate PCA. Both PCAs used 4 PCs and venetian blinds cross-validation.

Using the scores plots from each PCA (i.e. one of frequency domain features and the other of time domain features), the Euclidean distances between the scores of 5 pairs of audio files identified visually on each plot as being similar, and 5 identified visually as dissimilar were calculated (see below for more detail). These audio pairs were then ranked separately. One ranking used the Euclidean distance derived from frequency features. The other used Euclidean distance derived from the time domain features. A difference in the two rankings would suggest that the frequency domain data contains information that is not associated with the time domain data.

### Subjective audio assessment

The classification of similar and dissimilar sounds based on the frequency domain PCA was compared to subjective assessments by a panel of listeners. This was done to test if the features extracted from the audio signals were relevant to the subjective impression of the sound by human observers. This was a two-step process, as mentioned above, for Euclidean distance calculations. In the first step, based on the frequency domain PCA scores plot, a total of five pairs of audio files with similar PCA scores (i.e. with adjacent location in a scores plot) were visually identified on the scores plot. Then five pairs of sound files with dissimilar scores (i.e. pairs with the greatest Euclidean distance from each other) were identified on the scores plot. All pairs of similar and of dissimilar sounds were from different individual dogs. Thus a total of twenty audio samples were identified as being pairwise similar (5 pairs) or dissimilar (5 pairs) on the PCA scores plot. In the second step, 24 veterinary undergraduate students were asked to listen to these audio files. Similar and dissimilar pairs were played in sets of two pairs. Directly after hearing each set, the students were asked to select the most similar pair in the set. The result for each set of sound files was dichotomous because each assessor either agreed or did not agree with the classification determined quantitatively using frequency domain data and PCA. If the students agreed with the frequency domain PCA classification they would obtain a score of one, if they differed the score was zero. Total agreement between the student and the frequency domain PCA classification would result in a score of 5. The order and classification of the audio files were blinded to the students. They had no prior experience in Doppler ultrasound but had prior training in medical auscultation. Before being asked to assess the audio files, the students listened to three random examples of arterial Doppler recordings from the dataset, to introduce them to ultrasonographic Doppler sounds. The Fleiss-Kappa statistic was determined for all assessors combined to measure the interobserver variation. A one-sample t-test was performed, to examine the subjective scoring by the students. Because each question was binary, it is expected that there would be agreement in half (2.5 out of 5) occasions, if the human listeners made an unbiased guess. Therefore, an hypothesized mean of 2.5 was used for the t-test. The null hypothesis being that the students guessed completely at random and so the mean score was 2.5. The alternative hypothesis is that their mean was not 2.5, and that the student assessment was related to the PCA frequency domain classification. The significance level was set to p < 0.05.

## Ethics

All applicable international, national, and/or institutional guidelines for the care and use of animals were followed. All procedures performed involving animals and humans were in accordance with the ethical standards of the institution at which the studies were conducted. All analyses are presented anonymously. Informed written consent was obtained from the owner of each participating dog and all participating veterinary students. All parts of the study were approved by the Ethics and Administration Committee at the Department of Veterinary Clinical Sciences, Faculty of Health and Medical Sciences, University of Copenhagen (No. 2016–13).

## Results

A total of 22 dogs were included in the study. Their mean weight was 16.2 kg (median 12.3 kg) and the mean body condition score was 5.1 out of 9. The mean, median, standard deviation and minimum and maximum values for the time domain spectral features are summarized in Table 1.

**Table 1:** Time domain spectral Doppler features in 22 dogs.

	Median	Mean	SD	Min	Max
PSV_left_	139.95	143.36	19.23	112.28	184.02
PSV_right_	136.28	141.07	25.23	104.52	192.17
EDV_left_	35.98	37.20	13.27	22.08	71.15
EDV_right_	30.30	31.59	10.05	17.62	51.00
PI_left_	1.89	2.07	0.73	1.04	4.13
PI_right_	2.28	2.39	0.84	1.46	4.44
RI_left_	0.76	0.74	0.07	0.60	0.85
RI_right_	0.77	0.78	0.05	0.70	0.85

PSV = peak systolic velocity (cm/s), EDV = end diastolic velocity (cm/s), PI = pulsatility index, RI = resistive index. Values for these parameters were generated by software in the ultrasound machine during each scan. The table shows combined data for 22 dogs. SD = standard deviation.

A PCA including the time domain Doppler values for each dog (not shown) showed no evident grouping of the scores, i.e. there was a clear overlap between samples from dogs of different sex, age and size, as well as between the left and right sides of the neck and transducer direction. PC1 explained 66.15% of the variance and PC2 explained 13.03%.

**Figure 2:**
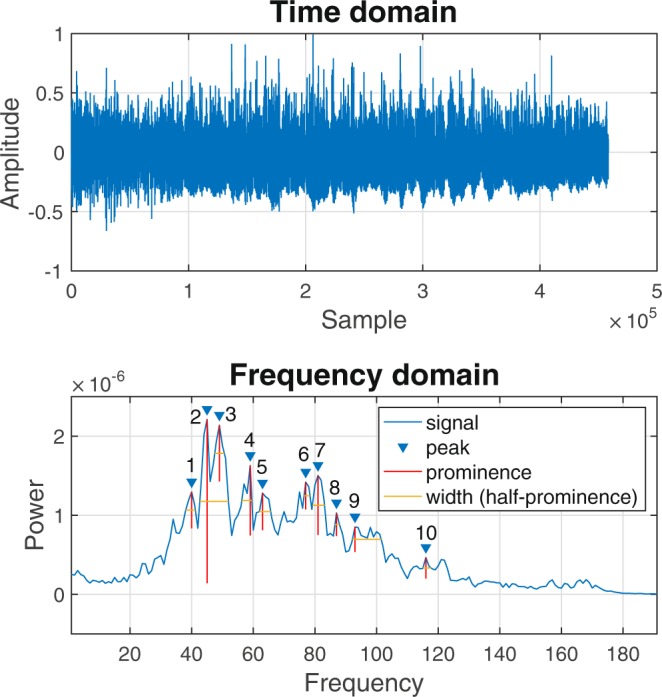
Example of an original Doppler audio signal in the time domain (top) and in the frequency domain, where the power spectral density estimate (PSDE) of the same signal is displayed after Fourier Transform (bottom). The PSDE has been processed so that high and low frequency signals (i.e., noise from instrumentation and adjacent tissues) are excluded. The ten most prominent peaks are automatically selected and numbered 1 to 10, according to their location on the x-axis (i.e. frequency). In further analysis, the location, prominence, peak and width at half prominence were used. In the lower plot frequency is displayed as bin number. The range of frequencies in the 200 bins displayed is approximately 800–16,000 Hz.

**Figure 3:**
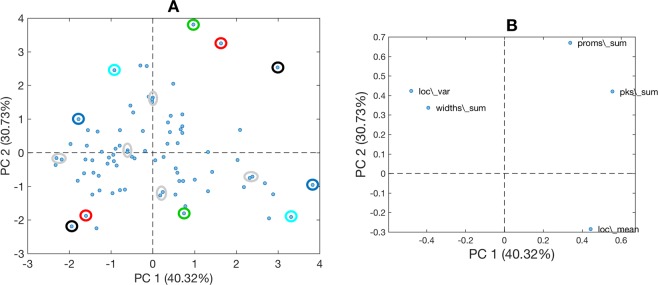
Principal component analysis. (**A**) Scores plot. (**B**) Loadings plot. Each score represents an audio file and each loading represents a frequency feature extracted from all of the audio files. Proximity in the scores plot indicates feature similarity, with regard to the variance explained by the principal components. The loadings represent the weighted importance of each feature. For example, loadings to the left on PC1 had a greater influence for scores to the left on PC1. The colored circles indicate sound files identified as dissimilar, where the same color indicates a dissimilar pair. The sound files enclosed in grey ellipses indicate similar pairs. On the plot axes, numbers in parentheses indicate the variance captured. PC = principal component.

The frequency domain features were extracted as described in the methods. An example of the time and frequency domain data as they appeared for one audio file in the processing software is given in Fig. 2. The PCA that includes the five frequency features extracted from the audio files is shown in Fig. 3. In this figure the 84 scores represent the audio recordings (four per dog, except for two dogs who only had two recordings) and the five loadings represent the frequency domain features. PC1 explained 40.32% of the variance and PC2 explained 30.73%. The scores plot again showed no clustering or grouping among dogs of different age, size, sex, body condition score, nor according to the side of the neck (left/right) scanned, the orientation of the transducer or the length of the sound files.

A total of 24 students in veterinary diagnostic imaging listened to the 5 sets of paired audio samples and identified the most similar pair in each set. Of the 24 students, 11 made subjective similarity classifications in agreement with the objective PCA classification for 5 out of 5 sets, 12 students were in agreement in 4 out of 5 sets and one student in 3 out of 5 sets. In total, 106 out of the 120 dichotomous classifications (88%) were in agreement with the quantitative model based on the frequency domain PCA. The Fleiss-Kappa test showed a substantial agreement between the subjective assessments made by the listening panel (Kappa value 0.657).

The average subjective score obtained by the students was 4.42 out of 5. This is significantly different to the hypothetical mean of 2.5 representing a 50% chance of agreement for each response (p < 0.001).

Pairs classified as similar or dissimilar were then ranked according to their Euclidean distance in the scores plot (Fig. 3). This rank order was different to the ranking based on the PCA of the time domain features. This suggests that information contained in the frequency domain differs from that obtained in the time domain.

## Discussion

This study confirms that audio fingerprinting can be applied to ultrasonographic Doppler signals from the common carotid arteries in dogs. The audio signals were indexed by extraction of multivariate frequency features that are readily quantified, and similarities and dissimilarities among the samples were identified using a machine learning method. The study also confirms that quantified frequency domain features reflect subjective non-expert perception of similarity among audio signals. In the majority of cases, the panel of listeners subjectively identified the same pairs of audio signals as being similar or dissimilar and thus agreed with the classification based on the quantitative model. This finding supplements a previous study where strong correlation between quantitative Doppler frequency features and the sound perception of expert listeners was reported. The study also showed that frequency domain features can be used to differentiate between samples that in the time domain appeared identical^11^. The complexity of the signals makes both subjective assessment of Doppler signals and automated analysis challenging. Nevertheless, the proposed audio fingerprinting method brings Doppler ultrasonography one step closer to automated analysis of blood flow and the proposed approach has potential value in computer-assisted analysis of Doppler signals^16^.

Medical ultrasound machines do not routinely record the sound produced by the Doppler shift. An immediate transformation of the Doppler shift information is performed by the ultrasound machine, to create the spectral curve which is displayed on the screen^7^. Based on this spectrum of a few cardiac cycles, specific time domain quantitative values are then extracted by the machine, thus representing a very short moment in time. These values and the spectral Doppler display are typically saved to the medical record. If in addition audio data were saved, these data could be analyzed in the frequency domain. This would allow identification of possible associations between frequency domain features and parameters available from other tests, such as ECG, blood pressure or other hemodynamic monitoring systems as suggested by other authors^17^.

Saving Doppler audio data together with analysis such as we have proposed would provide, over time, a large dataset. These audio data could be indexed using audio fingerprinting techniques. The features contained in any fingerprint should be a useful reflection of the content of the audio files and should be related to perceptually relevant cues. Such a database would be searchable, so that similar audio files could be retrieved. When files are linked to patients with known outcomes, this process of finding similar files could potentially assist clinicians in dealing with patients with unknown disease on initial presentation. If large numbers of well classified audio files become available, then supervised machine learning methods could be used, to generate prediction models that may assist in diagnosis samples for patients with unknown disease. Our study only included healthy individuals, and does not describe human data, but there is no reason to think experience would be otherwise with human arterial data. The principles of audio fingerprinting, indexing and searching are the same regardless of which audio signals that are used in the model.

Limitations of this study include the small population size. For robust use of machine learning applications, very large datasets are required^18^. Arterial diseases are rare in dogs and the common carotid artery is not routinely examined with spectral Doppler^19–21^. In humans however, the extra-cranial carotid arteries are often investigated with Doppler ultrasound, because of the risk of cardiovascular events and stroke following atherosclerosis^8,22^. The feature extraction method presented in this study is relatively simple. Other, more complex and elaborate methods of feature extraction have been described^10,23^. However, a strength of the relatively simple feature extraction described here is that it is robust against variations in insonated blood volume, heart rate, and insonation angle. These three parameters are important confounding factors in Doppler based hemodynamic studies. Notwithstanding the limitations of our study, our experience, even in a veterinary setting, might prompt interest in routine or research recording of audio signals from the Doppler studies of the carotid and other arteries. The data that accrued would allow case control studies to explore classification and prediction performance of automated and computer-assisted techniques including machine learning.

## Conclusion

In this study, we present a novel approach to analysis of arterial Doppler signals. By the use of an audio fingerprinting method based on PCA of frequency domain features in the audio signals, we identified Doppler audio files with similar and dissimilar properties. A subjective human evaluation of the audio files indicated good agreement with the automated identification of similar and dissimilar sounds from the same files. Our findings confirm that the quantitative frequency features contain perceptual information. The presented method is particularly relevant to applications where quantitative and objective spectral features from Doppler signals are desired, such as machine learning algorithms. This form of automated analysis is not available from current diagnostic ultrasound equipment, but we propose it could add value to future studies concerning blood flow. In addition the audio fingerprinting approach proposed here can be used to index and retrieve Doppler signal files from large databases. The agreement between the subjective (human) classification and the PCA classification suggests that it should be possible to query a database for Doppler sound files that are similar to those from a current but undiagnosed patient.

## Data Availability

Data and associated protocols are available to readers.

## References

[1] Pires IM (2018). Recognition of activities of daily living based on environmental analyses using audio fingerprinting techniques: A systematic review. Sensors.

[2] Bhatia S (2019). Systematic review of biometric advancement and challenges. Int. J. Electron. Eng..

[3] Lim S-C, Lee J-S, Jang S-J, Lee S-P, Kim MY (2012). Music-genre classification system based on spectro-temporal features and feature selection. IEEE Trans. Consum. Electron..

[4] Gavrovska A, Zajić G, Reljin I, Reljin B (2013). Classification of prolapsed mitral valve versus healthy heart from phonocardiograms by multifractal analysis. Comput. Math. Methods Med..

[5] Goto J (2015). Usefulness of a real-time bowel sound analysis system in patients with severe sepsis (pilot study). J. Artif. Organs.

[6] Sepehri AA, Kocharian A, Janani A, Gharehbaghi A (2016). An intelligent phonocardiography for automated screening of pediatric heart diseases. J. Med. Syst..

[7] Pellett AA, Kerut EK (2006). The Doppler velocity waveform. Echocardiography.

[8] Chuang S-Y (2016). Common carotid artery end-diastolic velocity is independently associated with future cardiovascular events. Eur. J. Prev. Cardiol..

[9] Übeyli ED, Güler I (2004). Spectral analysis of internal carotid arterial Doppler signals using FFT, AR, MA, and ARMA methods. Comput. Biol. Med..

[10] Latifoğlu F, Kara S, İmal E (2009). Comparison of short-time Fourier transform and eigenvector music methods using discrete wavelet transform for diagnosis of atherosclerosis. J. Med. Syst..

[11] Thuring A (2013). Operator auditory perception and spectral quantification of umbilical artery Doppler ultrasound signals. PLoS One.

[12] Maulik, D. *Doppler ultrasound in obstetrics and Gynecology*. (Springer, 2005).

[13] Pellerito, J. S. & Polak, J. *Introduction to vascular ultrasonography*. (Elsevier Saunders, 2012).

[14] Wold S, Esbensen K, Geladi P (1987). Principal component analysis. Chemom. Intell. Lab. Syst..

[15] Bro R, Smilde AK (2014). Principal component analysis. Anal. Methods.

[16] Latifoglu F, Şahan S, Kara S, Güneş S (2007). Diagnosis of atherosclerosis from carotid artery Doppler signals as a real-world medical application of artificial immune systems. Expert Syst. Appl..

[17] Herr MD (2010). A real-time device for converting Doppler ultrasound audio signals into fluid flow velocity. Am. J. Physiol. Heart Circ. Physiol..

[18] Mitchell, T. M. *The discipline of machine learning*. Vol. 9 (Carnegie Mellon University, School of Computer Science, Machine Learning Department, 2006).

[19] Lee K, Choi M, Yoon J, Jung J (2004). Spectral waveform analysis of major arteries in conscious dogs by Doppler ultrasonography. Vet. Radiol. Ultrasound.

[20] Svicero DJ, Doiche DP, Mamprim MJ, Heckler CMT, Amorim RM (2013). Ultrasound evaluation of common carotid artery blood flow in the Labrador retriever. BMC Vet. Res..

[21] Figurová M, Kulinová V (2017). Ultrasonographic Examination of Some Vessels in Dogs and the Characteristics of Blood Flow in These Vessels. Folia Vet..

[22] Liem MI (2017). Investigations of carotid stenosis to identify vulnerable atherosclerotic plaque and determine individual stroke risk. Circ. J..

[23] Dirgenali F, Kara S (2006). Recognition of early phase of atherosclerosis using principles component analysis and artificial neural networks from carotid artery Doppler signals. Expert Syst. Appl..

